# A network analysis of cofactor-protein interactions for analyzing associations between human nutrition and diseases

**DOI:** 10.1038/srep19633

**Published:** 2016-01-18

**Authors:** Marie Pier Scott-Boyer, Sébastien Lacroix, Marco Scotti, Melissa J. Morine, Jim Kaput, Corrado Priami

**Affiliations:** 1 The Microsoft Research – University of Trento Centre for Computational and Systems Biology (COSBI), Rovereto (TN), Italy; 2 Department of Mathematics, University of Trento, Italy; 3 GEOMAR Helmholtz Centre for Ocean Research Kiel, Kiel, Germany; 4 Nestlé Institute of Health Sciences, Lausanne, Switzerland

## Abstract

The involvement of vitamins and other micronutrients in intermediary metabolism was elucidated in the mid 1900’s at the level of individual biochemical reactions. Biochemical pathways remain the foundational knowledgebase for understanding how micronutrient adequacy modulates health in all life stages. Current daily recommended intakes were usually established on the basis of the association of a single nutrient to a single, most sensitive adverse effect and thus neglect interdependent and pleiotropic effects of micronutrients on biological systems. Hence, the understanding of the impact of overt or sub-clinical nutrient deficiencies on biological processes remains incomplete. Developing a more complete view of the role of micronutrients and their metabolic products in protein-mediated reactions is of importance. We thus integrated and represented cofactor-protein interaction data from multiple and diverse sources into a multi-layer network representation that links cofactors, cofactor-interacting proteins, biological processes, and diseases. Network representation of this information is a key feature of the present analysis and enables the integration of data from individual biochemical reactions and protein-protein interactions into a systems view, which may guide strategies for targeted nutritional interventions aimed at improving health and preventing diseases.

Malnutrition is a global problem that affects populations in low- and middle-income countries (LMICs) deficient in vitamins and minerals as well as individuals in developed economies and urban areas consuming excess calories with insufficient levels of some micronutrients^1^,^2^,^3^,^4^. Populations from Germany, the United States, and the United Kingdom were all reported to have deficient intakes of vitamin D along with folic acid in Germany, vitamins A and E in the United States, and vitamin E in the United Kingdom^1^. Micronutrient inadequacies (i.e. either too low or too high) may contribute to the development of age-related chronic diseases^5^,^6^. Extending the understanding of the role of micronutrients from reactions to physiological systems^7^ is of critical importance to address the maintenance of health, the needs of the malnourished individuals^8^, the promotion of maternal and fetal health and development^9^,^10^,^11^, and the requirements of at-risk groups such as the elderly^12^ and the obese^13^.

Current daily recommended intakes were usually established on the basis of the association of a single nutrient to a single, most sensitive adverse effect in the most susceptible subpopulation^14^. These recommendations are imperfect and may lead to misevaluation of micronutrient (in)adequacies because of (i) inter-individual variability in requirements due to the influences of age, gender, activity level, and metabolic and socioeconomic status (e.g.,^15^), (ii) the fact that plasma nutrient levels might not reflect tissue storage and needs^16^, and (iii) because they potentially neglect interdependent and pleiotropic effects of micronutrients on biological systems by focusing on single nutrients. Failure to consider system interactions may explain why epidemiological studies associating individuals or multi-micronutrient supplementation with morbidity of specific diseases continue to yield contradictory findings (e.g.,^17^,^18^,^19^).

Comprehensive databases linking multiple interactions of micronutrient (as cofactors) and components (proteins) to biological pathways and diseases are not available. To address this knowledge gap, data from multiple sources was integrated to create a comprehensive knowledgebase of cofactors, their protein interactions, and associated diseases. This dataset was represented as an integrative multi-layered network linking cofactors, cofactor-interacting proteins, biological processes, and diseases (Fig. 1). This approach builds on similar analysis of the human disease network in which diseases were connected if they shared genetic polymorphisms^20^ and the zinc proteome interaction network^21^. The integrative network analysis presented here aids in unraveling how micronutrient (in)adequacies can influence multiple biological processes, ultimately leading to health maintenance or disease progression.

## Materials and Methods

### Construction of the cofactor-protein network

The EBI CoFactor^22^, the Uniprot^23^, Expasy^24^ and the Metal MACiE^25^ databases were mined to identify human proteins that require cofactors. In the Uniprot database, both nutrients identified as cofactors as well as those specified as binding to a given protein were included. Non-specific cofactor requirements such as the case when either magnesium or manganese is required have been labeled as “metal”.

Manual curation of the data standardized the cofactor names, resulting in a single catalog of proteins associated with inorganic ions^25^ and organic and *in vivo* produced metabolites^22^. Vitamin A (retinol), Vitamin D (D3) and Vitamin E (alpha-tocopherol) are considered as transcriptional ligands or antioxidant molecules and are not in cofactor databases. The vitamin A/retinoic acid receptor RXR alpha and beta (RXRA and RXRB, respectively), vitamin D/vitamin D3 receptor (VDR), and Vitamin E and PXR were added to the combined list of proteins interacting with micronutrients.

Finally, information about genetic variants in the cofactor binding sites for some of the proteins in the catalog developed here was extracted from Uniprot database. The comprehensive dataset of proteins, the cofactor(s) with which they interact, and known binding site variants for those proteins are detailed in Supplementary Table S1.

The complete protein-cofactor dataset was represented as a network using Cytoscape 3^26^.

### Comparative analysis with the use of the protein-protein interaction network

#### Module detection

**Figure 2:**
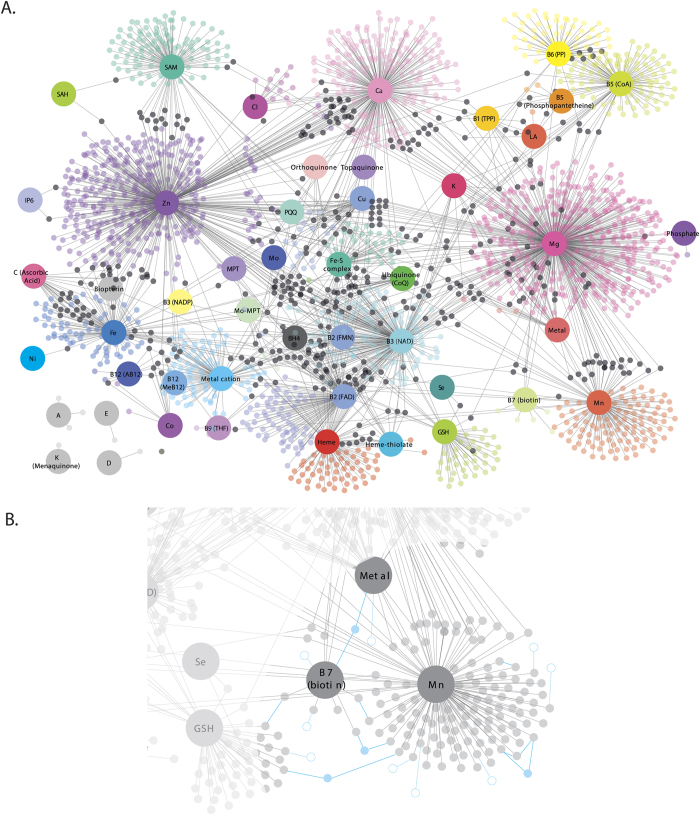
Cofactor-protein interaction network. (**A**) Larger nodes represent cofactors while smaller nodes represent proteins. The nodes are color-coded by cofactors where smaller black nodes represent proteins that interact with more than one cofactor. (**B**) Schematic representation of first-degree neighbors. Dotted nodes and edges represent first-degree neighbors interacting with only one cofactor-protein (not considered in analysis) while solid nodes and edges represent first-degree neighbors that are shared by at least two cofactor-interacting proteins (considered in analysis). Note that first-degree neighbors were not represented in (**A**) for ease of visualization. Abbreviations: AB12: Adenosylcobalamin, AMP: Adenosine monophosphate, BH4: Tetrahydrobiopterin, CoA: Coenzyme A, CoQ: Coenzyme Q, FAD: flavin adenine dinucleotide, Fe-S: Iron-Sulfur complex, FMN: Flavin mononucleotide, GO: Gene ontology, GSH: Glutathione, HPA: Human protein atlas, LA: Lipoic acid, LMIC: Low- and middle-income countries, MeB12: Methylcobalamin, MPT-Mo: Molybdopterin-Molybdenum, MPT: Molybdopterin, MTHF: Methyltetrahydrofolate, NAD: Nicotinamide adenine dinucleotide, NADP: Nicotinamide adenine dinucleotide phosphate, PP: Pyridoxal phosphate, PPI: Protein-protein interaction, PQQ: Pyrroloquinoline quinone, SAM: S-Adenosyl methionine, THF: Tetrahydrofolate, TPP: Thiamine pyrophosphate, VDR: Vitamin D receptor, and Vit: Vitamin.

We mapped the cofactor-interacting proteins to the protein-protein interaction (PPI) network from the Human Protein Reference Database (HPRD^27^) and extracted all cofactor-protein interactions. The first-degree neighbors (non-cofactor-interacting proteins) that were shared by at least two cofactor-interacting proteins were also extracted. Such selection of neighbor proteins was implemented to minimize the risk of diluting the focus on cofactor-interacting proteins with proteins at the periphery of the network. Instead, only protein neighbors that act as bridges between areas of the network comprising cofactors-interacting proteins were considered in the analysis (see Fig. 2B. for schematic representation).

The resulting network included 4,187 proteins (of which 1,183 are cofactor-interacting proteins) and 21,333 interactions, which consisted of 1,057 interactions between cofactor-interacting proteins, 8,935 interacting between cofactor-interacting proteins and their neighbors and 11,341 interactions between neighbors.

The module detection algorithm MCODE^28^ from the Cytoscape plugin clusterMaker (with parameters set to default) was then used to partition the network into modules. With such parameters, the algorithm groups only highly interacting proteins (i.e. removing singly-connected proteins from modules). Modules including more than 10 proteins were then further analyzed in order to identify over-representation of single cofactors and biological function enrichment for each module.

#### Analysis of protein-protein connectivity

The PPI network for the cofactor proteome was determined using the approach developed by Goh *et al*.^20^. The network was constructed including the interactions from HPRD, BIND and BioGrid. Data were extracted from the i2d database^29^ and self-loops and multiple interactions involving the same pairs of proteins were removed. The resulting network had 14,687 proteins and 149,435 interactions.

We then calculated the degree of connectivity of cofactor-interacting proteins in PPI network to identify the top 2% most connected proteins that were defined as hub proteins. It is generally assumed that such hub proteins have important biological roles^30^. Permutation tests were performed to evaluate if cofactor-interacting proteins were more connected than other, non-cofactor interacting proteins (the frequency of hubs found within non-cofactor interacting proteins was compared to that of cofactor-interacting proteins). This comparison was repeated 10,000 times.

The RNAseq data for 16 different tissues of the Human BodyMap 2.0 from Illumina database (GEO GSE30611) was used to evaluate if proteins interacting with the same cofactor had similar expression profiles. A total of 2,257 cofactor-interacting proteins (79.5% of all cofactor-interacting proteins) were present in the dataset. The Pearson’s product moment correlation coefficient across all tissues was calculated between all pairs of proteins (genes) that interact with a given cofactor. For each cofactor, the average of every correlation coefficient was then compared to the correlation of a random group of proteins (genes) of the same size. This was repeated 10,000 times in order to obtain a p-value for each cofactor. This analysis was run only for cofactors interacting with 10 or more proteins.

### Analysis of tissue-specific expression of cofactor-interacting proteins

Tissue-specific expression of cofactor-interacting proteins was evaluated using data extracted from the Human Protein Atlas (HPA) database^31^. This atlas provides information of antibody data from 82 cell types in 44 human tissues coupled with tissue-specific mRNA expression in 32 tissues. Cofactor-interacting proteins were classified into the following categories:

1. Tissue enriched: mRNA levels in one tissue at least five times higher than all other tissues,
2. Group enriched: mRNA levels of a group of 2 to 7 tissues at least five times those of all other tissues,
3. Tissue enhanced: mRNA levels in a particular tissue at least five times the average level in all tissues,
4. Expressed in all: mRNA detected in all tissues,
5. Mixed: detected in fewer than 32 tissues but not elevated in any tissue, or
6. Not detected.

Data mining with R package *RISmed* was conducted to identify the number of publications related to all cofactor-interacting proteins (genes) found for each HPA categories. We then compared the number of publications between categories to evaluate the possibility of publication bias (*Student t-test*).

### Construction of the cofactor-disease network

The DiseaseConnect database^32^, which associates genes with diseases, was used to link cofactor-interacting proteins to diseases. For the present analysis, data from OMIM (which included 3,644 genes and 4,299 diseases) and GWAS (which included 3,341 genes and 622 diseases) databases were used. The statistical significance of the representation of cofactor-interacting proteins in disease genes was evaluated with a hypergeometric test in comparison to a randomly selected set of genes (from HUGO database^33^).

A bipartite network (cofactor-disease) was constructed starting from cofactor-protein-disease interactions. A cofactor was associated with a disease if it interacted with a protein known to be linked to that disease. Edges were weighted to represent the number of proteins interacting with a given cofactor and associated with a disease. Cohesion and hierarchical structure of the cofactor-disease network were analyzed with nestedness. Nestedness is widely used in ecology for characterizing the hierarchical organization and asymmetry of interactions in bipartite networks (e.g., plant-animal mutualistic networks)^34^,^35^. It quantifies the paired overlap in the interaction patterns of species in ecological communities and its value ranges from 0 to 1. In the present case, this index was used to evaluate the relative importance of cofactors to a disease. Diseases with lower nestedness interact with more cofactors, while diseases with higher nestedness interact with fewer cofactors. In other words, nestedness index gives indication about the dependency of a given disease to cofactor availability. The nestedness index was calculated with the R package *bipartite*^36^ for all the diseases associated with more than 5 cofactor-interacting proteins (genes).

### Statistics, network analysis and gene enrichment analysis

All statistics were computed with R 3.0.1^37^. Network analysis was performed with the R packages *igraph*^38^ and *bipartite*. The DAVID Bioinformatics Resources 6.7 web service was employed to evaluate GO biological process and KEGG, Reactome and Biocarta pathway enrichment using a Benjamini and Hochberg significance cut-off of 0.05^39^.

## Results

### Cofactor-protein interaction network

Forty-nine (49) cofactors were retrieved from mining the EBI CoFactor, Uniprot, Expasy and Metal MACiE databases (refer to Fig. S1 for the classification of cofactors into their origins and to Fig. S2 for the overlap between information provided by these databases). A total of 2,840 unique cofactor-protein interactions between those 49 cofactors and 2,301 proteins were found. The complete list of cofactor-protein interactions, and the known genetic variants that alter protein’s cofactor binding site are listed in Supplementary Table S1. The resulting network representation of cofactor-protein interactions can be found in Fig. 2 where cofactor-interacting proteins (smaller nodes) were linked to their required cofactors (larger nodes).

We then investigated the biological roles played by cofactor-interacting proteins within protein complexes. With the module detection algorithm we identified 12 modules (including more than 10 proteins) of highly interacting proteins (see Table 1 and Supplementary Tables S2a and b). Most modules included proteins interacting with cofactors. Functional enrichment analysis revealed that proteins within modules are enriched for biological terms (GO) or pathways (KEGG, Reactome and Biocarta) with strong significance, thus confirming that the identified modules are grouping proteins that share functionally relevant interactions.

### Cofactor-interacting proteins topology in PPI network

Forty-six (46) of the 300 hub proteins found in the i2d database interact with cofactors. Permutation tests indicated that cofactor-interacting proteins do not have a significant tendency to be hub proteins in comparison to a random set of non cofactor-interacting proteins (*P* = 0.13, permutation test).

### Tissue-specific cofactor-interacting protein expression

Analysis of tissue-specific cofactor-interacting protein expression revealed that 1,271 (44.8%) of these proteins are expressed in all tissues, 236 (8.3%) proteins are mixed, 133 (4.7%) proteins are group-enriched, 362 (12.7%) proteins are tissue-enhanced, 236 (8.3%) proteins are tissue-enriched (summarized in Table 2 and detailed in Supplementary Table S3), and 63 (2.2%) proteins are not detected. Comparison with randomly selected sets of proteins revealed that cofactor-interacting proteins are more often expressed in all tissues (*P* < 0.001, permutation test). These results might be explained by publication bias since widely expressed genes would be represented more often in protein-cofactor interaction knowledgebases. However, further analysis was performed to test for publication bias and confirmed the difference in expression in all tissues between cofactor and non-cofactor enzymes. Interestingly, 34% of proteins enriched in adrenal glands, 26% of the proteins enriched in liver, and 25% of those enriched in pancreas require cofactors. Lower percentages of cofactor-interacting proteins per tissue-enriched proteins are found in bone marrow (9%) cerebral cortex (9%), testis (5%), and heart (6%).

Furthermore, RNAseq data from 16 different human tissues was used to assess if proteins interacting with a given cofactor have similar tissue expression profiles. This analysis revealed that proteins interacting with vitamins B1, B2, B3, B6, glutathione, S-adenosylmethionine, heme, ubiquinone, Fe-S complex and Mg are more often co-expressed across tissues than what is randomly expected (p-value < 0.01).

### Cofactor-disease interaction network

**Figure 3:**
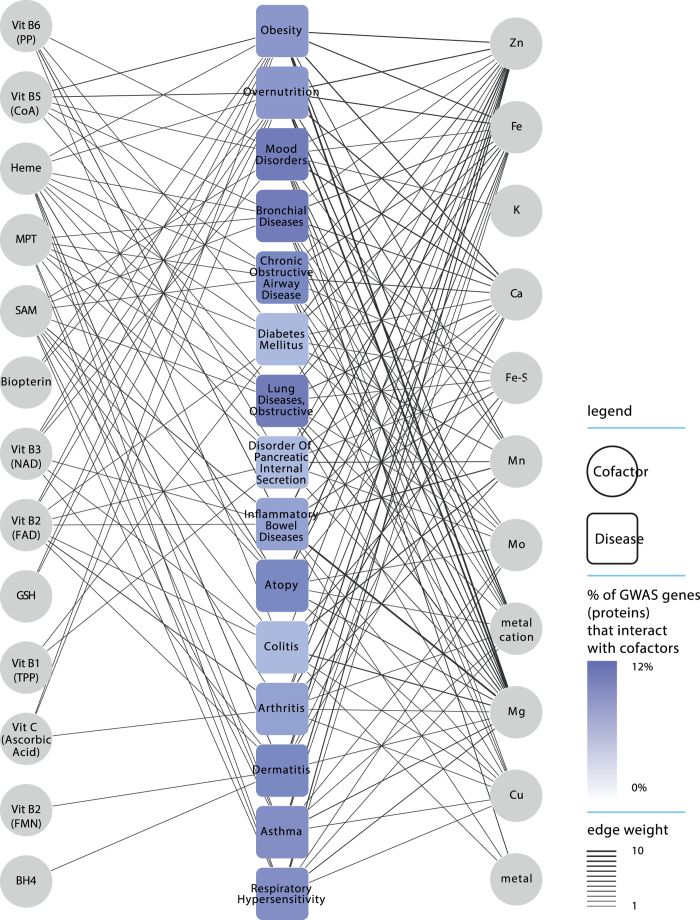
Cofactor-disease network. The cofactors (circles) are linked to a GWAS disease (squares) if protein(s) associated with that given disease interact with the target cofactors. Diseases are color-coded according the percentage of GWAS proteins that interact with cofactors and ranked according to nestedness (ascending order from top to bottom). Edges are weighted by the number of GWAS proteins that require a given cofactor.

The GWAS database contains 379 diseases that are associated with at least one gene coding for a cofactor-interacting protein (which is 60.9% of the 622 diseases in the database; see Fig. 3 and Supplementary Table S4). We calculated nestedness index (ranging from 0 to 1) to evaluate the diversity of the cofactors with which disease-proteins (genes) interact. The diseases with the lowest nestedness (i.e. diseases that interact with the most diverse set of cofactors) are obesity, overnutrition, mood disorders, bronchial diseases, chronic obstructive airway disease, and diabetes mellitus (Fig. 3). On average, 10% of proteins (genes) associated with those diseases interact with at least one cofactor. In the case of obesity, which is the disease with the lowest nestedness index (index of 0), disease-related proteins (genes) interact with 26 different cofactors (i.e. representing 53% of all studied cofactors).

The OMIM database contains 1,354 diseases that are linked to at least one gene coding for a cofactor-interacting protein (31.4% of a total of 4,299 diseases; see Supplementary Table S4). In addition, 573 of the 2,301 cofactor-interacting proteins (24.9%) were linked to at least one disease, a statistically significant enrichment (P < 0.001, hypergeometric test). The OMIM diseases with the lowest values of nestedness are linked to nutritional status in which cofactors adequacy could potentially influence disease initiation, progression, and/or outcome. For example, if we consider the diseases associated with more than 50 genes, four conditions (i.e. deficiency diseases, nutritional disorders, malnutrition and mitochondrial diseases) showed a high percentage of cofactor-interacting proteins: 40% of proteins involved in deficiency diseases, 40% of nutritional disorders proteins, 41% of malnutrition proteins, and 39% of mitochondrial disease proteins interact with cofactors (Supplementary Table S4).

## Discussion

A century of research focusing on individual reactions and related pathways has produced detailed biochemical maps of intermediary metabolism. While these maps are foundational for understanding the range of biological processes that produce health or disease, metabolism is a complex system integrating processes of different sub-networks. The activity of the sub-networks may be affected by multiple inputs. We performed an integrative analysis on the cofactors required for many protein- and protein-mediated reactions. All of these cofactors are derived directly or through metabolism from naturally occurring dietary chemicals and can thus be influenced by dietary habits and interventions.

Developing this integrative knowledgebase required a multistep process of combining information on proteins and their cofactor interactions from multiple databases, followed by network analysis. The requirement to integrate information about cofactor-protein interactions from the different databases is understandable because some of these databases are specialized for certain classes of cofactors. Indeed, the EBI CoFactor database is specialized on organic cofactors while the MACiE database focuses on metal ions. Integrating this knowledge warranted the creation of a unified and comprehensive dataset including all micronutrients and micronutrient-derived cofactors as described in this report. The representation of this knowledge into a network is a key feature of the analysis presented here since it facilitates the understanding of the multiple and broad interactions between cofactors and proteins (Fig. 2). The associations of the cofactor-interacting proteins (genes) to diseases (Fig. 3) may provide strategies for targeting nutritional interventions to modulate complex phenotypes.

Analysis of the cofactor-interacting protein network augmented for selected first-degree neighbors (refer to *Materials and Methods* section) revealed that cofactor-interacting proteins are involved in a large variety of fundamental biological functions (Table 1) that could be involved in the development of complex disease phenotypes. Moreover, more cofactor-interacting proteins than other proteins are expressed in many tissue types (Table 2). Proteins that interact with certain cofactors, most notably organic vitamins, tend to be co-expressed in all tissues, which could result from similarly regulated ADME (i.e. absorption, distribution, metabolism and excretion) processes. These data may be used to more critically analyze and test Ames’ triage theory^5^, which states that in the context of nutrient deficiencies, micronutrients (and by implication, their cofactors) would be preferentially used in reactions and processes that ensure short-term survival (e.g., energy production) over those involved with long-term survival (e.g., DNA repair). Although our data cannot strictly test this hypothesis, it can serve to estimate the importance of different micronutrients in short- and long-term survival associated processes. For instance, cofactor-interacting proteins were over-represented (P ≪ 0.001, hypergeometric test) in genes (proteins) involved in DNA repair (GO:0006281). Cofactor-interacting proteins involved in genome integrity required significantly more Mg, Fe-S complex and THF (vitamin B9) than randomly selected cofactor-interacting proteins (P < 0.01, permutation test). It could thus be postulated that deficiencies in one or more of these cofactors could significantly impact DNA repair and thus hamper long-term survival. This would however need to be validated experimentally but recent publications showed that DNA damage was minimized in cell cultures under elevated folate concentrations^40^ and suggested a critical role of Fe-S complexes in long-term coordination of DNA replication and repair^41^.

Overt nutrient deficiencies are increasingly rare, at least in developed and many LMICs, while nutrient inadequacies of varying degree are more likely to occur^1^,^42^. These inadequacies may result from normal aging^43^ or from metabolic deregulations that underlie age-related or obesity-related disorders^18^,^44^ that may affect micronutrient absorption, transport, or utilization. However, nutrient inadequacies may also be observed in otherwise healthy individuals and be the result of genetic variants affecting nutrient (cofactor) absorption, tissue-specific distribution, and/or utilization in biochemical reactions. A possible and likely scenario to explain such phenotypes is that genetic variants in cofactor transporters or in cofactor binding-sites may affect tissue absorption or distribution. Variants may also directly or indirectly alter cofactor-protein binding affinity^25^ and, in some cases, biochemical parameters that affect substrate utilization within the cofactor interactome. One or a combination of these elements could modify nutrient bioavailability and requirements, explain inadequacies and resulting metabolic alterations. The K_M_ mutant theory put forward by Ames *et al*. furthers this hypothesis and postulates that increasing levels of micronutrients – by means of lifestyle modifications or micronutrient interventions – could compensate genetic variants lowering the affinity constants (increased K_M_) of some enzymes^45^.

To extend this concept, the cofactor-interacting proteins were associated with diseases from the OMIM database. Unsurprisingly, top diseases were those related to nutritional aspects such as malnutrition and nutrient adequacy. Interestingly, mitochondrial disease was also associated with a large number of proteins interacting with cofactors. For example, important proteins from the mitochondrial complex I-II-III (e.g., CYC1, NDUFA1-10, NDUFS1-2-3-7, NDUFV1-2, SDHA/B, COQ6 and PDSS1) and those involved in energy metabolism (PDHA1 and ACAD9/VL) interact with many cofactors including magnesium, zinc, and NAD. However, OMIM diseases are primarily Mendelian (i.e. single gene) diseases and inborn errors of metabolism, which may thus be weakly influenced by nutrition in comparison to complex diseases, such as those listed in GWAS databases. Complex phenotypes result from the interplay between multiple low-penetrant polymorphisms and environmental factors.

We mined the GWAS database to identify genes coding for cofactor-interacting proteins associated with complex phenotypes. GWAS disease-genes (proteins) that interact with the most diverse cofactors (low nestedness indices) were associated with obesity and overnutrition (both sharing the same gene set). Functional analysis of the GWAS cofactor-genes revealed significant GO biological function for purine and nitrogen metabolism (*ADCY3-9, ARG, GCH1, ATP12A, KMO*). In addition, the genes identified by GWAS for these diseases mostly interact with metal cofactors with similar molecular weight and oxidation number (i.e. Mg^2+^, Mn^2+^, Zn^2+^, and Ca^2+^) (although different in sizes and hydration spheres). Alterations in absorption, distribution, or binding mechanisms of those metals could affect tissue-specific bioavailability and deregulate energy metabolism. In addition, variations in cofactor binding sites such as those found in GTP cyclohydrolase (GCH1; Uniprot P30793, variants in amino acids at position 141, 144 and 212) could alter its cofactor affinity (K_M_) and requirements. This enzyme is the rate-limiting enzyme in tetrahydrobiopterin (BH_4_) biosynthesis and modulation of its activity would in turn influence BH_4_ related pathways such as nitric oxide metabolism and endothelial function, and the one-carbon pool by folate pathway (see Supplementary Table S1).

Recent observations showed benefits of normalization by supplementation of micronutrient inadequacies often associated with over-consumption of Western-type diets. A number of cardiometabolic markers were positively modulated in obese individuals provided 8 weeks of a multi-nutrient supplementation^46^. These results support our observation that obesity-related proteins interact with a large array of diverse cofactors affecting different subsystems such as pathways and processes involved in and contributing to cardiovascular health. Hence, obese individuals may potentially benefit from targeted improvement of micronutrient bioavailability. Interestingly, the authors of the study suggest that the improvements in cardiometabolic markers could result from improvements of mitochondrial function, which is, as noted herein, associated with a large number of cofactor-interacting proteins.

**Figure 4:**
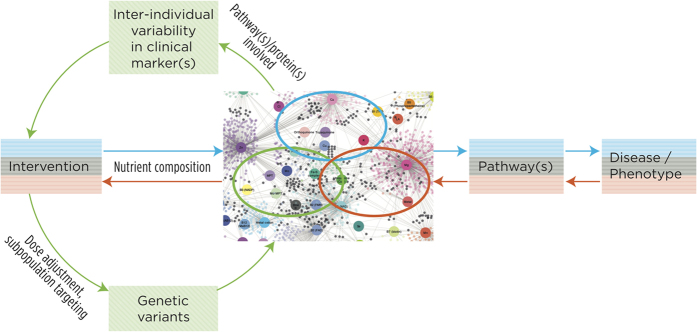
Applicability of the cofactor-interacting protein network. Schematic representation of three potential applications of the cofactor-interacting protein network. A first path (blue) can be followed in order to identify the proteins, biological pathway(s) (circled in blue in the network) and clinical phenotype(s) or disease that should be modulated by an intervention on the basis of its nutrient composition. A second path (red) following the reverse approach in which cofactor-interacting proteins (circled in red in the network) involved in biological pathway(s) or disease of interest can be mapped onto the network in order to identify the cofactors – and by extension the nutrients – that would need to be targeted by a nutrient-based intervention in order to modify said phenotype(s) of interest. A third path (green) can rely on the network to investigate the potential origins of inter-individual variability in response to a nutritional intervention by investigating genetic variants in cofactor-interacting proteins involved in proteins and pathway(s) (circled in green in the network) linked to clinical markers of interest.

The networks and dataset presented here could be used to unravel the influence of micronutrient (in)adequacies on biological processes and constitute part of the knowledgebase supporting interventions aimed at promoting health, and preventing / reversing many adverse phenotypes and diseases associated to cofactor-interacting proteins. First, the subset of proteins (circled in blue in Fig. 4) that should be modulated by a dietary intervention (on the basis of its micronutrient composition) can be identified and assessed in the cofactor-interacting protein network. By contributing to improve the understanding of how biological pathways are targeted by the dietary intervention this could unveil possible effects on clinical phenotype or disease (blue path in Fig. 4). Second, the cofactor-interacting proteins involved in biological pathways or disease of interest can be mapped onto the network (circled in red in Fig. 4). Such pathways can be used to identify which cofactors – and by extension which nutrients – have to be targeted by the intervention to modify the (clinical) phenotype of interest (red path in Fig. 4). Third, genetic variants in cofactor-interacting proteins that are involved in pathways linked to clinical markers and show inter-individual response variability to a nutritional intervention (circled in green in Fig. 4) can be investigated. This can contribute to better understand the origin of such variability and the information gained from such study can be used for tailoring genotype-specific interventions or identifying subpopulations with better odds of responding positively to the dietary intervention (green path in Fig. 4). These premises are, however, based on the assumption that improvements of nutrient bioavailability ameliorate cofactor-interacting protein (enzymatic) function. This would need to be further investigated since information to that effect is not yet always available.

Furthermore, the paths depicted in Fig. 4 can also be followed in a tissue-specific manner in cases were the (patho)physiological condition of interest involves only a single or a few tissue(s). In such instances, the network would first be pruned to keep only proteins expressed in all tissues (i.e “housekeeping” proteins) and those enriched and/or enhanced in the tissue(s) related to the condition investigated. Such strategy may improve investigations of the impact of cofactor availability or nutrient interventions by reducing the potential interference of proteins (and associated pathways) unrelated to the condition being investigated. Similarly, tissue and/or condition-specific regulation of protein activity could be accounted for in building a context-specific network^47^. However, knowledge regarding post-translational regulation might be too sparse to be fully exploited at the moment.

The limitations of the compilation of cofactor-protein interactions and the network analysis are the availability of data in cofactor databases and publication bias. Nevertheless, the results presented here provide an integration of data from disparate sources to create a more comprehensive, systems knowledgebase for micronutrient and cofactor processes that alter metabolism. The development of this cofactor-protein interaction knowledgebase provides an approach to better study and explain the effects of multivitamin and mineral intake on different processes, in different tissues, and in different metabolic states and diseases.

## Conclusions

“Tuning-up” metabolism^48^ to optimize health and delay or prevent disease^49^ will likely not be possible with single nutrient interventions. The systems approach described here shows the overlapping metabolic processes that often require multiple cofactors from different dietary components (e.g., metal ions versus vitamins). These data and results are being extended to assess how population and individual allele frequencies may influence specific biological processes identified in this analysis and how dietary intakes could be mapped through the knowledgebase developed in the present project to allow prediction of nutrient needs. The goal of these efforts is to contribute to a better molecular understanding of the consequences of nutritional inadequacies. The integrated database and network analysis reported here represents an important step that will be the foundations onto which targeted nutritional interventions aimed at improving micronutrient status will be based in efforts to normalize impaired biological functions.

## Additional Information

**How to cite this article**: Scott-Boyer, M. P. *et al*. A network analysis of cofactor-protein interactions for analyzing associations between human nutrition and diseases. *Sci. Rep.* **6**, 19633; doi: 10.1038/srep19633 (2016).

## Supplementary Material

Supplementary Figures

Supplementary Table 1

Supplementary Table 2a

Supplementary Table 2b

Supplementary Table 3

Supplementary Table 4

## Figures and Tables

**Figure 1:**
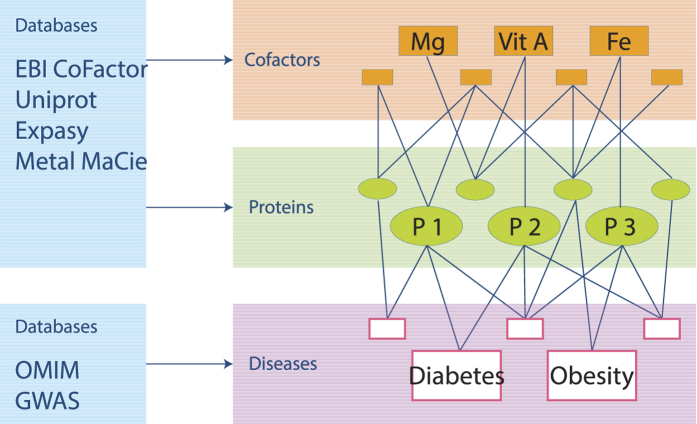
Conceptual representation illustrating the construction of the cofactor-disease network. A third level of information was added to the cofactor-protein network to include interaction with disease genes. A cofactor is linked to a disease if they share the same cofactor-interacting protein(s).

**Table 1:** Modules detected in the network of cofactor-interacting proteins and their first-degree neighbors.

Module	Number of Proteins	Number of cofactor-protein (%)	Cofactor	Main Biological Functions and/or Pathways
1	10	1 (10.0%)	Mg	(ribosomal)RNA processing and ribosome biogenesis
2	19	2 (10.5%)	Mg, Vit B6 (PP)	Positive regulation of transcription and RNA metabolic process.
				HIV infection.
3	83	19 (22.9%)	Ca, Mg, Zn, SAM, B5 (CoA)	Regulation of transcription, cell proliferation and apoptosis.
				Cell surface signal transduction. Response to hormone (insulin) stimulus
				Erb and PDGF signaling.
4	31	2 (6.5%)	Mn, Zn	Cell surface signal transduction, Phosphate metabolic process, Regulation of apoptosis.
				B and T cell receptor signaling
5	109	26 (23.9%)	Ca, Cu, Mg, Mn, SAM, Vit B5 (CoA), Zn, metal	Regulation of macronutrient metabolism, cell differentiation, apoptosis, angiogenesis and TGFß signaling.
				Pathway in colorectal and pancreatic cancer.
6	110	26 (23.6%)	Ca, Mg, SAM, Vit A, Vit B2 (FAD), Vit B9 (THF), Zn	Regulation of apoptosis, RNA metabolism, transcription factor activity, and protein kinase activity. Response to stress. NOD/Toll receptors signaling.
7	110	15 (13.6%)	Ca, Fe, Mg, Mn, SAM, Zn, metal, metal cation	Regulation of transcription, immune response, phosphorylation, and apoptosis.
8	46	10 (21.7%)	Fe-S complex, Mg, Zn, GSH, Vit B2 (FAD)	Regulation of transcription, phosphorylation, JNK and MAPK activity. Insulin signaling pathway
9	12	5 (41.7%)	Mg, Mn	Regulation of apoptosis, JNK and MAPKKK activity
10	85	15 (17.6%)	Ca, Mg, Mn, Zn, SAM, metal	Phosphate metabolic process.
				Regulation of apoptosis.
11	12	4 (33.3%)	Ca, Mg, Zn, metal	–
12	25	9 (36.0%)	Mg, Zn, metal, Vit B3 (NAD)	Regulation of phosphate metabolism, protein kinase activity, cell proliferation and differentiation.
				TGFß signaling, TCA cycle, signaling by BMP and diabetes pathways.

Details of number of proteins, number (percentage) of cofactor-interacting proteins, cofactor interactions and main biological functions (GO) and pathways (KEGG hsa, Reactome and Biocarta) annotation per module.

**Table 2:** Tissue-enriched cofactor-interacting proteins.

Tissue	Number of tissue-enriched cofactor-interacting protein	Total number of tissue-enriched proteins^1^	Percentage (%)
Endometrium	2	4	50
Adrenal gland	13	38	34
Liver	45	172	26
Pancreas	11	44	25
Small intestine	1	4	25
Adipose tissue	4	21	19
Prostate	4	21	19
Gallbladder	1	6	17
Ovary	1	6	17
Placenta	15	86	17
Thyroid gland	4	23	17
Tonsil	1	6	17
Skeletal muscle	17	111	15
Esophagus	6	43	14
Duodenum	1	8	12
Spleen	1	8	12
Salivary gland	5	45	11
Stomach	3	28	11
Bone marrow	8	85	9
Cerebral cortex	29	318	9
Kidney	6	68	9
Skin	7	97	7
Heart muscle	2	33	6
Lung	1	17	6
Testis	47	999	5
Fallopian tube	1	60	2

^1^From Human Proteome Atlas^31^.
