# Supplementary material for: A network analysis of cofactor-protein interactions for analyzing associations between human nutrition and diseases
Source: Sci Rep. 2016 Jan 18;6:19633. doi: 10.1038/srep19633 (PMC4726080; doi:10.1038/srep19633)
Supplement: Supplementary Figures [file srep19633-s1.pdf]

A network analysis of cofactor-protein interactions for analyzing associations between human nutrition and diseases

Marie Pier Scott-Boyer[1*+], Sébastien Lacroix[1+], Marco Scotti[1,3], Melissa J. Morine[1], Jim Kaput[4] and Corrado Priami[1,2]

[1] The Microsoft Research – University of Trento Centre for Computational and Systems
Biology (COSBI), Rovereto (TN), Italy
[2] Department of Mathematics, University of Trento, Italy
[3] GEOMAR Helmholtz Centre for Ocean Research Kiel, Kiel, Germany
[4] Nestlé Institute of Health Sciences, Lausanne, Switzerland

* Corresponding author, scottboyer@cosbi.eu
[+] These authors contributed equally to the work

Supplementary Information (SI)

Table S1. Cofactor-protein interactions and genetic variants in cofactor-binding sites. List of cofactor-protein interactions from all mined databases and genetic variants in cofactor-binding sites from the Uniprot database.

Table S2a. Gene ontology annotation results for each module detected in the cofactor-interacting proteins network.

Table S2b. Biological pathway enrichment results for each module detected in the cofactor-interacting proteins network.

Table S3. Tissue expression of cofactor-interacting proteins.

Table S4. Cofactor-disease interactions. OMIM and GWAS diseases associated with genes encoding for cofactor-interacting proteins.

Figure S1. Cofactor classification.

Figure S2. Overlap of cofactor-interacting proteins between the four different databases.

Figure S3. Per cofactor representation of the degree of connectivity of cofactors-interacting proteins.

Cofactors

Inorganic

- Copper (Cu)
- Iron (Fe)
- Magnesium (Mg)
- Manganese (Mn)
- Molydenum (Mo)
- Nickel (Ni)
- Zinc (Zn)
- Cobalt (Co)
- Potassium (K)
- Calcium (Ca)
- Chloride (Cl)
- Selenium (Se)
- Phosphate

Metal complex

- Fe-S complex

Organic

Vitamin

- Thiamine pyrophosphate [Thiamine (B1)]
- Flavin adenine mono/dinucleotide (FMN/FAD)[Riboflavin (B2)]
- NAD+ and NADP+ [Niacin (B3)]
- Coenzyme A/Phosphopantetheine [B5]
- Pyridoxal phosphate [Pyridoxine (B6)]
- Biotin (B7)
- Tetrahydrofolic acid [Folic acid (B9)]
- Methylcobalamin/AdenosylCobalamine (B12)
- Ascorbic acid (C)
- Menaquinone (K)

Transcription factor ligands

- Retinol(A): RXR-RAR
- D3:VDR
- E: PXR

Non-vitamin

- In box non-vitamin box
- Biopterin
- Glutathione (GSH)
- Heme
- Heme-thiolate
- Lipoic Acid (LA)
- Molybdopterin (MPT)
- Orthoquinone
- Pyrroloquinoline quinone (PQQ)
- S-Adenosyl methionine (SAM)
- S-Adenosyl-L-homocysteine (SAH)
- Tetrahydrobiopterin (BH4)
- Topaquinone
- Ubiquinone (CoQ)

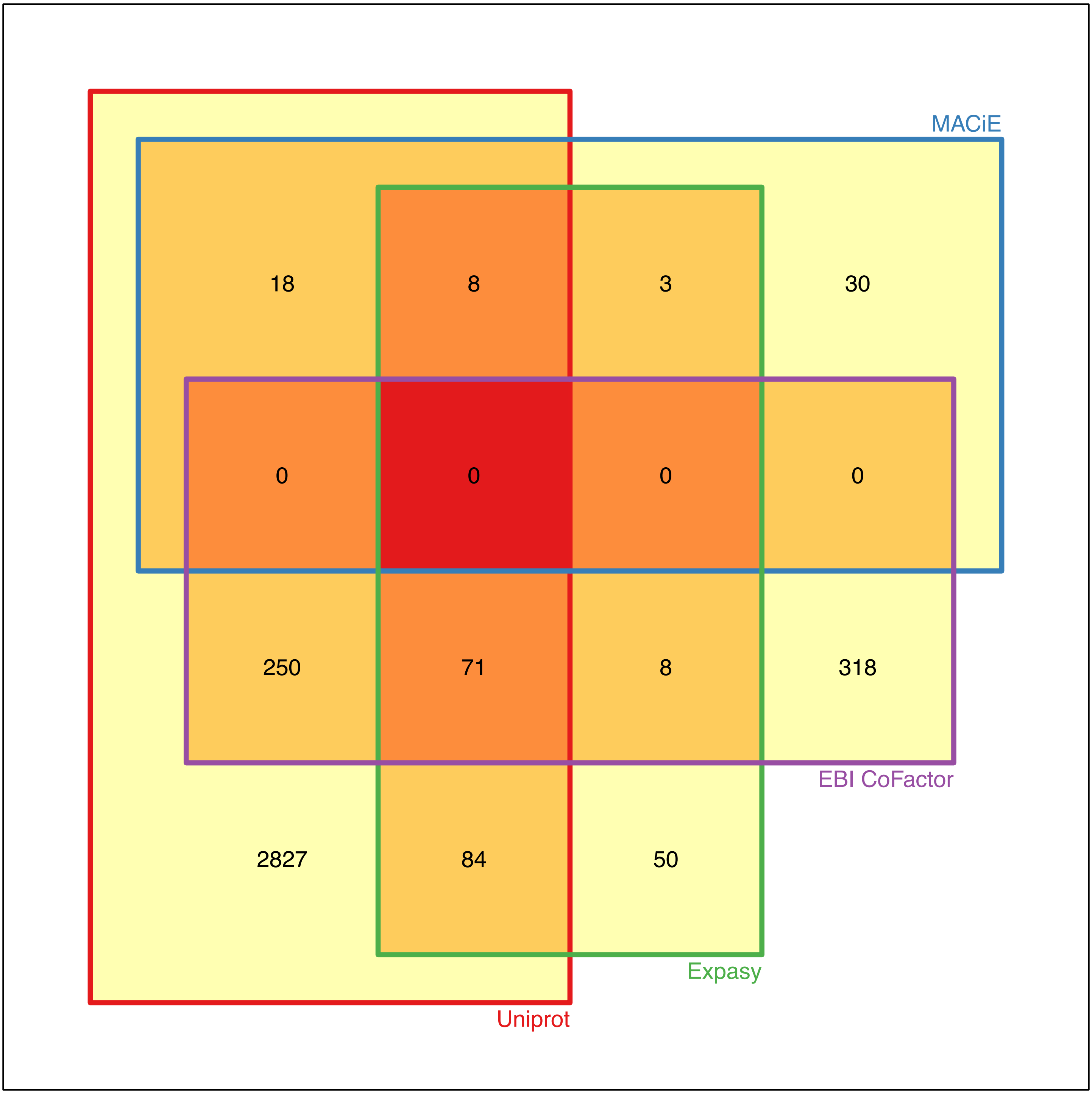
MACiE
18
8
3
30
0
0
0
0
250
71
8
318
EBI CoFactor
2827
84
50
Expasy
Uniprot

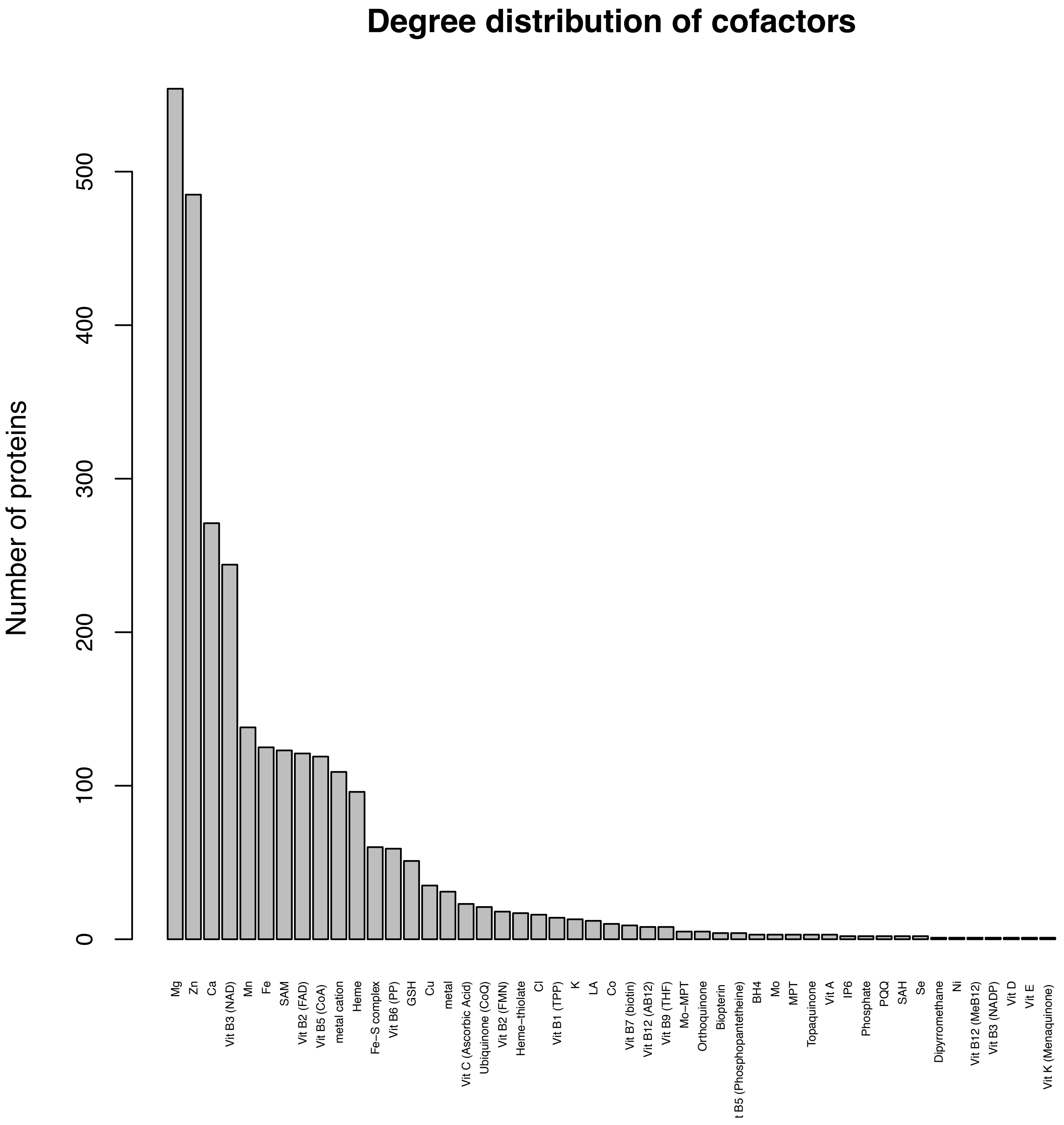
Degree distribution of cofactors
Number of proteins
0
100
200
300
400
500
Mg
Zn
Ca
Vit B3 (NAD)
Mn
Fe
SAM
Vit B2 (FAD)
Vit B5 (CoA)
metal cation
Heme
Fe−S complex
Vit B6 (PP)
GSH
Cu
metal
Vit C (Ascorbic Acid)
Ubiquinone (CoQ)
Vit B2 (FMN)
Heme−thiolate
Cl
Vit B1 (TPP)
K
LA
Co
Vit B7 (biotin)
Vit B12 (AB12)
Vit B9 (THF)
Mo−MPT
Orthoquinone
Biopterin
t B5 (Phosphopantetheine)
BH4
Mo
MPT
Topaquinone
Vit A
IP6
Phosphate
PQQ
SAH
Se
Dipyrromethane
Ni
Vit B12 (MeB12)
Vit B3 (NADP)
Vit D
Vit E
Vit K (Menaquinone)
